# Comparison of Single Use and Traditional Negative Pressure Wound Therapy Devices in Lower Extremity Ulcers: A US Real‐World Evidence Analysis of NetHealth Data

**DOI:** 10.1111/iwj.70756

**Published:** 2025-09-04

**Authors:** Alison Garten, Leo M. Nherera, Rodney Lindsay

**Affiliations:** ^1^ Charlotte Limb Preservation and Diabetic Foot Centre Charlotte North Carolina USA; ^2^ Smith + Nephew Inc Fort Worth Texas USA; ^3^ Carrollton Regional Medical Centre Carrollton Texas USA

**Keywords:** diabetic foot ulcer, lower extremity ulcer, single‐use negative pressure wound therapy, traditional negative pressure wound therapy, venous leg ulcer

## Abstract

Annually, 49 million people worldwide are impacted by lower extremity ulcers (LEUs). Diabetic foot ulcers (DFUs) and venous leg ulcers (VLUs) are the most common LEUs. Negative pressure wound therapy (NPWT) has emerged as an effective intervention for complex wounds, offering numerous favourable wound healing outcomes. The objective of this study was to evaluate the effectiveness of single‐use NPWT (sNPWT) versus traditional NPWT (tNPWT) for wound closure in LEUs. Real‐world data was obtained from the US‐based Net Health outpatient database between January 2014 and October 2020 and included patients with LEUs (DFU or VLU) who had been treated with sNPWT or tNPWT. The rate of wound closure and time to wound closure were selected as endpoints. The wound closure rate was significantly higher for all LEUs (*p* = 0.039), VLUs alone (*p* = 0.003) and there was no difference for DFU (*p* = 0.90) that were treated with sNPWT versus tNPWT. The median time to wound closure was significantly shorter for sNPWT (114 days) compared to tNPWT (140 days, *p* < 0.01). Using sNPWT was associated with significantly higher wound closure rates and shorter time to wound closure. The results provide supportive evidence for using sNPWT for LEUs, demonstrating the opportunity to directly decrease the clinical burden of LEUs on patients. Subgroup analysis revealed a significant difference in wound closure rates for VLU, while no significant difference was observed for DFU. The overall LEU findings may be attributed to differences in the mechanisms of action between the two devices.

**Summary**

- This manuscript presents a US‐based real‐world evidence study comparing single‐use negative pressure wound therapy (sNPWT) with traditional NPWT (tNPWT) in the treatment of lower extremity ulcers (LEUs), including diabetic foot ulcers (DFUs) and venous leg ulcers (VLUs). Using data from the Net Health outpatient database (2014–2020), the study evaluated wound closure rates and time to closure in a matched cohort. Results showed that sNPWT achieved significantly higher closure rates in all LEUs and VLUs, but no difference for DFUs, and was associated with a significantly shorter median healing time. These findings suggest sNPWT may reduce the clinical and economic burden of LEUs by accelerating wound healing, especially in VLUs, although DFUs remain more challenging to treat.

## 1 Introduction

It is estimated that as many as 49 million people worldwide are impacted by lower extremity ulcers (LEUs) annually [1]. Diabetic foot ulcers (DFUs) and venous leg ulcers (VLUs) are the most common types of LEUs found on the feet (80%) and legs (60%–90%), respectively [1, 2]. LEUs reduce patient quality of life (QoL) through prolonged disability, pain and restriction on social functioning and often result in lower extremity amputation or premature death [3, 4, 5]. In fact, DFU patients have a 2.5‐fold increased risk of mortality compared to diabetic patients without DFUs [6, 7].

Ulcer‐related costs represent a substantial economic burden on healthcare systems globally [8, 9, 10]. The cost of care for patients with recurring VLUs may be up to three times higher than for patients without recurring VLUs [11]. Additionally, a prospective database study published in 2018 stated that the overall mean cost of care per DFU episode can be as high as $50 000 per patient, depending on ulcer severity [8]. Inpatient and outpatient care are major contributors to ulcer‐related treatment costs; wound care measures that decrease healing time and prevent even a minor proportion of wounds from progressing to inpatient hospital care or requiring extra outpatient treatment could favour associated costs [8, 9, 11, 12].

Numerous guidelines exist for the management of LEUs, including compression therapy, wound debridement, manual lymphatic drainage, off‐loading, infection and/or biofilm control, and evaluation for venous insufficiency, all of which are often considered standard of care (SoC) [13, 14, 15]. More recently, negative pressure wound therapy (NPWT) has emerged as an effective intervention, with numerous favourable wound healing outcomes in various complex wounds [16, 17, 18]. NPWT generally involves applying a wound dressing and sealing the area with an adhesive film; a controlled negative pressure or vacuum is then applied, ranging from −50 to −125 mmHg [19, 20]. NPWT may benefit LEUs as it can reduce the number of dressing changes compared to SoC, manage drainage, prevent repeated wound exposure, and provide the necessary stimulation to ulcers in slower healing patients to decrease wound closure time [21, 22, 23, 24, 25].

PICO™ (single‐use NPWT [sNPWT]; Smith + Nephew Medical Ltd., Hull, United Kingdom) and 3M™ V.A.C.® (traditional NPWT [tNPWT]; Solventum, San Antonio, Texas) are both NPWT devices with differing modes of action [26, 27, 28]. The tNPWT device has a default pressure setting of −125 mmHg, which can be varied by ±25 mmHg increments, is changed every 2–3 days, and has over 25 years of clinical use [26, 29, 30, 31]. sNPWT is a small, lightweight system that applies a constant −80 mmHg of pressure to the wound, that lasts up to 7 days, and has been in clinical use for over 10 years [32, 33]. Both devices have demonstrated superior clinical outcomes compared to SoC in LEUs [21, 22, 32, 34]. A randomised controlled trial (RCT) demonstrated sNPWT was superior to tNPWT across numerous wound healing outcomes, including wound depth and time to wound closure, and an economic evaluation conducted in 2020 in the United States (US) found sNPWT was more likely to be cost‐effective than tNPWT, from a healthcare payer perspective [35, 36]. However, a head‐to‐head comparison of the effectiveness of the two devices for wound closure when treating LEUs is yet to be performed [35]. This study uses real‐world evidence to directly compare wound closure rate and median time to healing LEUs treated with sNPWT and tNPWT.

## 2 Methods

### 2.1 Data Source

Data was from the US‐based Net Health, an outpatient wound clinic electronic medical records (EMR) database between January 2014 and October 2020, and was provided via CSV files (Net Health, Pittsburgh, PA). Data tables were imported into SAS 9.4 (SAS Institute Inc., Cary, North Carolina, US) and organised by product, with a set of files for each product name. Lengths of some variables were changed to prevent truncation in the combined datasets. No extra cleaning procedures were performed before applying the eligibility criteria. Since all patient records were de‐identified and the data complied with the Health Insurance Portability and Accountability Act (HIPAA), obtaining informed consent was not required. This study was exempt from Institutional Review Board (IRB) approval since it was conducted from commercially available, retrospective and routinely captured EMR data.

### 2.2 Summary of Identification of Cases

Patient data were obtained from the following NetHealth data tables using pattern matching: *npwt, physician orders, treatmentnotes, skinsubstitutes* and *otherprocedures*. sNPWT visits were identified by matching \bPICO\b OR PICO7, where \b indicates a word boundary. Similarly, tNPWT visits were identified using \bVAC\b OR ACTIVAC. Eligibility criteria were applied to the full population. Patients with LEUs (defined as a DFU or VLU) in whom NPWT was initiated within 60 days of the first recorded wound assessment between January 2014 and October 2020 were included. Patients who had wounds treated with both sNPWT and tNPWT, more than one wound treated with NPWT, or had insufficient wound assessment information, such as a wound area that was either zero or missing at the start of NPWT, were excluded from the study.

For each ulcer, all visits after the first NPWT treatment visit were identified (including visits unrelated to NPWT), and wound assessment data for each visit were obtained. Non‐NPWT visits following the initiation of NPWT were included as it is understood that although NPWT may aid wound closure, it is not necessarily used up to the point of closure itself.

### 2.3 Summary of Patient Demographics and Matching

Patient characteristics were compiled to allow matching between the two groups; these included the age and gender of the patient and the region where the ulcer was treated. Comorbidities recorded at the baseline NPWT visit were compiled from the *medicalhistory* and *complaintandsymptom* Net Health data tables. The tNPWT and sNPWT cohorts were matched using propensity score matching, and dummy dichotomous variables were generated when necessary to facilitate their inclusion in the matching process.

Propensity score matching was performed to minimise confounding and improve comparability between the sNPWT and the tNPWT cohorts by balancing baseline characteristics, thereby approximating the conditions of randomisation. We used logistic regression to calculate the propensity score for each patient in the comparison groups. A greedy matching algorithm with a 1:1 ratio (*k* = 1) paired each treated patient with the closest unmatched control based on propensity score. A calliper of 0.2, which measures the maximum allowed distance between matched pairs, was chosen, ensuring that only good‐quality matches are made [37]. The variables listed in Table S1 were used in the matching process. The balance between the groups was defined to be achieved when the absolute values of the standardised differences of the matching variables were no greater than 0.1.

### 2.4 Endpoints

Two analyses were conducted for the rate of wound closure: one for all ulcers (LEUs: both DFUs and VLUs; analysis 1) and one for separate DFUs and VLUs (analysis 2). The wound area was calculated as the length multiplied by the width and depth of the wound.

The analysis included all ulcers (LEUs: both DFUs and VLUs) for time to wound closure. Although visit dates were not included in the data, the number of days since the first assessment was recorded, and therefore, the timepoint in days from the initial NPWT visit could be calculated. Wound closure time was defined as the number of days since NPWT initiation at which the ulcer area was 0, and the variable *finalwoundstatus* had the status ‘*Resolved*’. Where subsequent ulcer recurrence was evident from the data, closure was not deemed to have occurred.

Ulcers were considered lost to follow‐up if the last recorded visit did not meet the following requirements: ulcer area of 0 and the variable *finalwoundstatus* was ‘*Resolved*’.

### 2.5 Summary of Statistical Analysis

A non‐parametric survival analysis was performed to generate each group's Kaplan–Meier estimator of the survival function. This enabled comparison between the two groups using the Tarone‐Ware test. To test for proportional hazards, the Kaplan–Meier plots were visually inspected, log(−log) plots were constructed, Schoenfeld residuals were plotted, and the supremum test for the proportional hazards assumption was used. These methods indicated that the proportional hazards assumption was not violated, and therefore, it was appropriate to use a Cox proportional hazards analysis to compare the two groups. An exploratory parametric survival analysis was conducted to investigate whether this approach could be relevant for comparing the groups. A sensitivity analysis was performed, which limited the results to one ulcer per patient. All analyses were conducted using SAS 9.4.

## 3 Results

### 3.1 Matching and Cases

There were 315 LEUs identified that were treated with sNPWT and 2659 LEUs treated with tNPWT. Before matching, ulcer initial depth, area, and type were not well matched; the mean baseline area was noticeably lower for ulcers treated with sNPWT (9.00 cm^2^) than for those treated with tNPWT (25.21 cm^2^) (Table 1). Baseline ulcer depth was also lower for ulcers in the sNPWT group (4.08 mm) compared to ulcers in the tNPWT group (10.46 mm), and treatment with the device was, on average, started later for ulcers treated with sNPWT (21.39 days) compared to ulcers treated with tNPWT (14.37 days) (Table 1). There was a higher proportion of DFUs in the tNPWT group (83.57%) compared to the sNPWT group (45.71%), but a higher proportion of VLUs in the sNPWT group (54.29%) compared to the tNPWT group (16.43%) (Table 1).

**TABLE 1:** Unmatched patient and ulcer characteristics for LEU patients treated with sNPWT or tNPWT.

	sNPWT	tNPWT	SMD (*p*)
Ulcer characteristics			
Total ulcers, *n* (%)	315 (100)	2659 (100)	
DFU, *n* (%)	144 (45.71)	2222 (83.57)	
VLU, *n* (%)	171 (54.29)	437 (16.43)	
Continuous variables: SMD (95% CI) *p* value			
Baseline wound surface area (cm^2^), mean	9.00	25.21	−1.62 (−17.38, −15.04) *p* < 0.0001
Baseline wound depth (mm), mean	4.08	10.46	−0.64 (−7.55, −5.21) *p* < 0.0001
Days before NPWT started, mean	21.39	14.37	0.7 (5.85, 8.19) *p* < 0.0001
Age at first visit, mean	66.95	60.13	0.68 (5.65, 7.99) *p* < 0.0001
BMI continuous, mean	31.76	32.27	−0.05 (−1.68, 0.66) *p* = 0.39
Categorical variables SMD (*p* value)			
Smoking status			
Missing/other, *n* (%)	104 (33.00)	499 (18.76)	0.180 (*p* < 0.0001)
Non‐smoker, *n* (%)	97 (30.90)	1090 (41.00)	
Smoker, *n* (%)	114 (36.10)	1070 (40.24)	
BMI category			
Normal/overweight, *n* (%)	152 (48.20)	1308 (49.20)	−0.09 (*p* = 0.16)
Obese, *n* (%)	95 (30.2)	856 (32.20)	
Severe obese, *n* (%)	53 (16.8)	394 (14.80)	
Underweight/missing, *n* (%)	15 (4.7)	101 (3.8)	
Diabetes mellitus			
No, *n* (%)	172 (54.63)	1348 (50.68)	−0.08 (0.31)
Yes, *n* (%)	143 (45.37)	1311 (49.32)	
Hypertension			
No, *n* (%)	150 (47.77)	1304 (49.03)	0.03 (*p* = 0.62)
Yes, *n* (%)	165 (52.23)	1355 (50.97)	
Obesity			
No, *n* (%)	279 (88.44)	2285 (85.93)	−0.08 (*p* = 0.25)
Yes, *n* (%)	36 (11.56)	374 (14.07)	
Arthritis			
No, *n* (%)	241 (76.38)	2200 (82.73)	0.16 (*p* = 0.0057)
Yes, *n* (%)	74 (23.62)	459 (17.27)	
Cancer			
No, *n* (%)	282 (89.45)	2519 (94.75)	0.19 (*p* = 0.0011)
Yes, *n* (%)	33 (10.55)	140 (5.25)	
Hyperlipidemia			
No, *n* (%)	229 (72.85)	2062 (77.55)	0.11 (*p* = 0.15)
Yes, *n* (%)	86 (22.15)	597 (22.45)	
Gender			
Female, *n* (%)	125 (39.68)	773 (29.07)	0.22 (*p* ≤ 0.0001)
Male, *n* (%)	188 (59.68)	1874 (70.48)	
Missing, *n* (%)	2 (0.63)	12 (0.45)	
Geographic region			
Mid‐West, *n* (%)	73 (23.10)	230 (10.20)	0.192 (*p* < 0.0001)
North‐East, *n* (%)	40 (12.70)	211 (9.32)	
South, *n* (%)	139 (44.20)	1062 (47.02)	
West, *n* (%)	63 (20.00)	756 (33.46)	

*Note:* Covariates between matching cohorts with *p* value > 0.05 or absolute SMD < 0.10 were considered well‐balanced. The *p*‐value provided and smd is applicable to the whole category because when comparing accross groups (sNPT vs tNPWT) you are testing whether the distribution of the entire varible differes between groups, the individual rows complement each other additing upto 100% (in underlined).

Abbreviations: BMI, body mass index; DFU, diabetic foot ulcer; LEU, lower extremity ulcer; NPWT, negative pressure wound therapy; SMD, standardised mean difference; sNPWT, single‐use negative pressure wound therapy; tNPWT, traditional negative pressure wound therapy; VLU, venous leg ulcer.

Following matching, there were 304 LEUs in the sNPWT and tNPWT groups, including 142 (46.71%) DFUs and 162 (53.29%) VLUs in both (Table 2). Ulcer characteristics, including mean baseline area (sNPWT; 9.29 cm^2^ vs. tNPWT; 10.86 cm^2^) and baseline ulcer depth (sNPWT; 4.12 mm vs. tNPWT; 4.27 mm), were similar between the groups (Table 2). Furthermore, all other patient baseline characteristics, such as smoking status, body mass index (BMI), and diabetes mellitus, matched well as determined by the *p* value > 0.05 or absolute SMD < 0.1 (Table 2) [37].

**TABLE 2:** Matched patient and ulcer characteristics for LEU patients treated with sNPWT or tNPWT.

	sNPWT	tNPWT	SMD (*p*)
Ulcer characteristics			
Total ulcers, *n* (%)	304 (100)	304 (100)	
DFU, *n* (%)	142 (46.71)	142 (46.71)	
VLU, *n* (%)	162 (53.29)	162 (53.29)	
Continuous variables: SMD (95% CI) *p* value			
Baseline wound surface area (cm^2^), mean	9.29	10.86	0.16 (−3.16, 0.02) *p* = 0.05
Baseline wound depth (mm), mean	4.12	4.27	−0.01 (−1.74, 1.44) *p* = 0.85
Days before NPWT started, mean	21.58	17.05	0.45 (2.94, 6.12) *p* < 0.0001
Age at first visit mean	66.58	66.53	0 (−1.54, 1.64) *p* = 0.95
BMI continuous, mean	31.83	31.65	0.02 (−1.41, 1.77) *p* = 0.83
Categorical variables SMD (*p* value)			
Smoking status			
Missing/other, *n* (%)	98 (32.24)	88 (28.94)	0.041 (*p* = 0.67)
Non‐smoker, *n* (%)	95 (31.25)	98 (32.24)	
Smoker, *n* (%)	111 (36.51)	118 (38.82)	
BMI category			
Normal/overweight, *n* (%)	148 (48.68)	151 (49.67)	0.03 (*p* = 0.71)
Obese, *n* (%)	93 (30.59)	93 (30.59)	
Severe obese, *n* (%)	49 (16.12)	47 (15.45)	
Underweight/missing, *n* (%)	14 (4.61)	13 (4.20)	
Diabetes mellitus			
No, *n* (%)	179 (58.88)	183 (60.20)	0.03 (*p* = 0.73)
Yes, *n* (%)	125 (41.12)	121 (39.80)	
Hypertension			
No, *n* (%)	147 (48.36)	142 (46.71)	−0.03 (*p* = 0.75)
Yes, *n* (%)	157 (51.64)	162 (53.29)	
Obesity			
No, *n* (%)	273 (89.80)	261 (85.86)	−0.12 (*p* = 0.16)
Yes, *n* (%)	31 (10.20)	43 (14.14)	
Arthritis			
No, *n* (%)	241 (79.28)	238 (78.29)	−0.02 (*p* = 0.83)
Yes, *n* (%)	63 (20.72)	66 (21.71)	
Cancer			
No, *n* (%)	275 (90.46)	280 (92.11)	0.06 (*p* = 0.49)
Yes, *n* (%)	29 (9.54)	24 (7.89)	
Hyperlipidemia			
No, *n* (%)	220 (72.37)	225 (74.01)	0.04 (*p* = 0.68)
Yes, *n* (%)	84 (27.63)	79 (25.99)	
Gender			
Female, *n* (%)	119 (39.14)	119 (39.14)	0 (*p* = 1)
Male, *n* (%)	183 (60.20)	183 (60.20)	
Missing, *n* (%)	2 (0.66)	2 (0.66)	
Geographic region			
Mid‐West, *n* (%)	73 (24.01)	69 (22.70)	0.019 (*p* = 0.97)
North‐East, *n* (%)	38 (12.50)	37 (12.17)	
South, *n* (%)	131 (43.09)	132 (43.42)	
West, *n* (%)	62 (20.39)	66 (21.71)	

*Note:* Covariates between matching cohorts with *p* value > 0.05 or absolute SMD < 0.10 were considered well‐balanced. The *p*‐value provided and smd is applicable to the whole category because when comparing accross groups (sNPT vs tNPWT) you are testing whether the distribution of the entire varible differes between groups, the individual rows complement each other additing upto 100% (in underlined).

Abbreviations: BMI, body mass index; DFU, diabetic foot ulcer; LEU, lower extremity ulcer; NPWT, negative pressure wound therapy; SMD‐standardised mean difference; sNPWT, single‐use negative pressure wound therapy; tNPWT, traditional negative pressure wound therapy; VLU, venous leg ulcer.

### 3.2 Rate of Wound Closure

**FIGURE 1:**
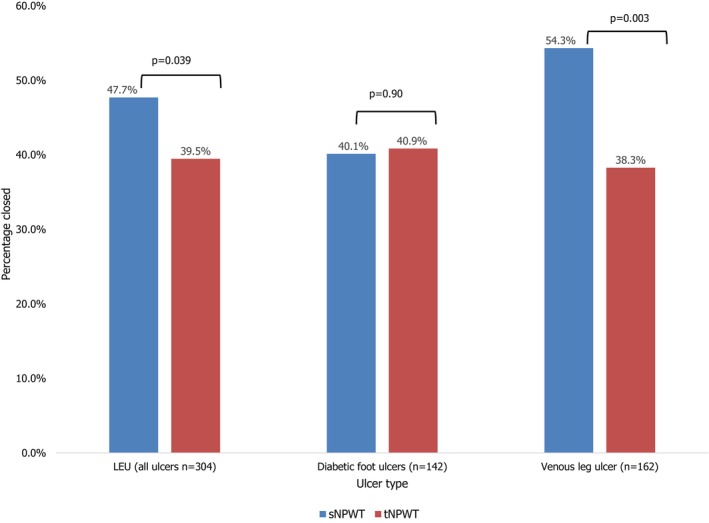
The rate of wound closure is shown as percent closed (%) when using sNPWT or tNPWT for LEUs (all ulcers), DFUs, and VLUs. DFU, diabetic foot ulcer; LEUs, lower extremity ulcers; sNPWT, single‐use negative pressure wound therapy; tNPWT, traditional negative pressure wound therapy; VLU, venous leg ulcer.

The wound closure rate was significantly higher for all LEUs that were treated with sNPWT compared to tNPWT (sNPWT: 47.70% closed; tNPWT: 39.47% closed; odds ratio [OR]: 1.45; 95% CI [1.02–2.03]; *p* = 0.039) and VLUs (sNPWT: 54.32% closed; tNPWT: 38.27% closed; OR: 2.19; 95% CI [1.23–2.99]; *p* = 0.003), but was comparable for DFUs (sNPWT: 40.14% closed; tNPWT: 40.85% closed; OR: 0.97; 95% CI [0.60–1.56]; *p* = 0.90) (Figure 1).

### 3.3 Time to Wound Closure

**FIGURE 2:**
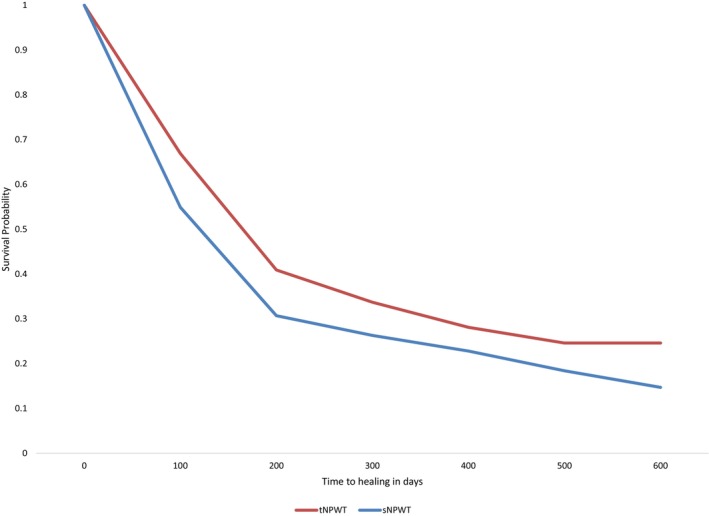
Kaplan–Meier curve demonstrating time to wound closure across all LEUs when using sNPWT or tNPWT. LEUs, lower extremity ulcers; sNPWT, single‐use negative pressure wound therapy; tNPW, traditional negative pressure wound therapy; VLU, venous leg ulcer.

A Kaplan–Meier curve demonstrated that the median days to closure for all LEUs was 114 days for the sNPWT group and 140 days for the tNPWT group, as seen in Figure 2 and Table 3. The time to wound closure was significantly shorter for sNPWT compared to tNPWT (Tarone‐Ware test for equivalence: *p* < 0.05; Cox proportional hazards model: *p* < 0.01). Table 3 presents the hazard ratios for wound closure across different time periods, all of which showed statistically significant differences.

**TABLE 3:** Time to wound closure and hazard ratios at different time points for LEU patients treated with sNPWT or tNPWT.

*t*	Time (days)	tNPWT Number not healed (*P*)	tNPWT Wounds healed	sNPWT Number not healed (*P*)	sNPWT Wounds healed	HR—at time *t* (sNPWT vs. tNPWT)
0	0	304 (1)	0	304 (1)	0	—
1	100	203 (0.67)	101	167 (0.50)	137	0.67 (0.52–0.87) *p* = 0.002
2	200	124 (0.41)	180	93 (0.31)	211	0.76 (0.62–0.93) *p* = 0.007
3	300	102 (0.34)	202	80 (0.26)	224	0.81 (0.67–0.98) *p* = 0.03
4	400	85 (0.28)	219	69 (0.23)	235	0.86 (0.72–1.03) *p* = 0.11
5	500	75 (0.25)	229	56 (0.18)	248	0.83 (0.69–0.99) *p* = 0.042
6	600	75 (0.25)	229	45 (0.15)	259	0.73 (0.61–0.87) *p* = 0.001

Abbreviations: HR, hazard rate; *P*, proportion of wounds not healed; sNPWT, single‐use negative pressure wound therapy; *t*, time; tNPWT, traditional negative pressure wound therapy.

### 3.4 Likelihood of Wound Closure

The sensitivity analysis was limited to patients with a single ulcer, resulting in 246 LEU patients in each NPWT group. The results demonstrated that the likelihood of wound closure was significantly higher for LEUs that were treated with sNPWT compared to tNPWT (OR: 1.62; Wald 95% confidence interval [CI]: [1.10, 2.38]; *p* = 0.038).

## 4 Discussion

This retrospective study directly compares tNPWT and sNPWT using real‐world data from the Net Health database to determine their effect on wound healing closure outcomes in comparable LEUs. When comparing tNPWT and sNPWT, the use of sNPWT was associated with a higher rate of wound closure in all LEUs and VLUs and a comparable rate of wound closure in DFUs. Additionally, sNPWT was associated with a shorter time to wound closure in all LEUs compared to tNPWT.

The clinical, economic and social burden of LEUs on the healthcare system in the US is high [9, 38, 39, 40]. LEUs are commonly seen in elderly populations. When combined with the current aging population, as well as the increasing rates of obesity and diabetes mellitus, chronic LEUs are predicted to become an increasingly persistent public health problem [3, 41, 42, 43, 44]. Various studies have confirmed that NPWT leads to improved clinical outcomes compared with SoC, providing an innovative approach to improving outcomes in various wound types, including LEUs [16, 17, 18, 45, 46, 47]. NPWT provides compressive forces through negative pressure; this accelerates wound healing by reducing local exudate and edema, stimulating tissue granulation, and promoting wound contraction [30, 32, 45, 48, 49]. The current study allowed for a direct comparison of two commonly used NPWT devices, sNPWT and tNPWT, in treating LEU for improved clinical outcomes. sNPWT is indicated for use in chronic wounds with a smaller area and low‐to‐moderate exudate levels and in cases requiring early discharge. In contrast, tNPWT is often used for chronic wounds with delayed or impaired healing, including DFUs and VLUs, with high exudate production [33, 50]. Additionally, tNPWT and sNPWT can be considered complementary and used sequentially as treatment progresses [33, 50].

This study suggests that the rate of wound closure is higher when using sNPWT compared to tNPWT. The results indicated that the wound closure rate was significantly higher for all LEUs and VLUs when using sNPWT compared to tNPWT and comparable in DFUs. This study also demonstrated that those treated with sNPWT healed significantly faster across all ulcers than those treated with tNPWT. Considering that the primary clinical goal in treating LEUs is to ensure rapid and complete healing, these results suggest that using sNPWT could provide an opportunity to achieve this clinical goal through reduced wound healing time compared to tNPWT [51, 52]. This improvement in wound healing could improve patient QoL by positively impacting physical, psychological and social domains typically affected by chronic ulceration [4, 53]. Patients with LEUs often suffer from problems like pain and sleep disturbance, all of which contribute to disruption of daily life and feelings of depression [54, 55]. The emotional impact on patients may be further exacerbated by emotional distress from body image disturbance [53].

Their differing mode of action may explain the significant difference in clinical outcomes between the two devices [26, 28, 56]. The sNPWT device has a high moisture vapour transmission film top layer, with reports of up to 85% of the fluid, such as exudate, removed via evaporative loss [28, 57]. The tNPWT device does not have a high moisture transmission layer and instead has a foam dressing [26, 29]. This difference in technology may lead to variation in wound moisture and exudate levels and, consequently, affect clinical outcomes [58]. However, international consensus acknowledges that tNPWT continues to play a role in managing certain wound types [59]. Specifically, wounds suitable for tNPWT device consideration are those where the size, depth and exudate volume exceed the management capabilities of an sNPWT system [59].

The non‐significant difference in wound closure rates for DFUs between the two devices could be due to the complex pathogenesis and unpredictable, refractory nature of this ulcer type [60]. Therefore, NPWT devices could have a smaller impact on DFU wound healing than expected, so differences in outcomes across devices are more challenging to observe.

An increased wound healing rate has both direct and indirect benefits. Patients would directly benefit from a shorter wound healing time as it would reduce patient disability and wound‐related pain at rest [61]. Increased pain for the patient has also been reported during dressing changes [62]. Furthermore, changing wound dressings is an element of ulcer care that can often be the responsibility of a family member, which can lead to patients feeling like a burden [53]. Therefore, the decreased frequency of dressing changes associated with using sNPWT compared to tNPWT could also contribute to improved patient QoL through reduced pain and increased independence [61, 62]. In general, sNPWT devices are smaller and more discreet than tNPWT devices and, therefore, more portable and less cumbersome; this could lead to improved ease of use, freedom of movement and increased patient compliance [32, 52]. Additionally, sNPWT is used predominantly in an outpatient setting; this is less common in tNPWT, which is more often used for inpatient care [33]. sNPWT could provide greater patient autonomy, flexibility and convenience.

Chronic LEUs represent a significant financial challenge for the healthcare sector and patients. In 2014, the US's annual costs associated with DFUs and VLUs were approximately $9–13 and $15 billion, respectively [39, 63]. More recently, the annual direct medical costs associated with VLUs were estimated to be $4.9 billion in the US in 2019 [9]. Improved wound healing rates and faster time‐to‐heal could also lead to a lower number of inpatient, outpatient and home care visits, which are significant contributors to expenditures related to wound care [11, 12, 62]. Additionally, patients with DFUs have significantly reduced work ability, including a decreased capacity to perform physical demands and increased time lost from work, compared to both diabetic patients without ulcers and healthy controls (*p* < 0.0001) [64]. Patients have also reported economic stressors associated with DFUs, including loss of income or changes to employment from the inability to work, demonstrating that the economic burden of LEUs extends to patients themselves [53]. Accelerated wound healing may reduce disability and enhance mobility in patients with LEUs, lowering societal financial burdens like work absenteeism and lost productivity. Further economic benefits may be derived from the price difference between the two devices. sNPWT is associated with a lower daily cost than tNPWT, owing to the sNPWT device including one pump and two dressings to last 7 days, whereas tNPWT has to be changed every 2–3 days [26, 28, 35]. Less frequent dressing changes allow sNPWT to be more cost‐effective than tNPWT potentially [35].

Overall, this work may inform and help guide clinical practice for treating LEUs. This study highlights the importance of comparative effectiveness research. It directly compares two different NPWT devices to assist clinicians and policy makers when making decisions to improve clinical outcomes in LEUs and reduce the associated healthcare costs [52, 65].

## 5 Limitations

This study is subject to the common drawbacks of administrative discharge data, such as missing data and improper or incomplete coding [66]. Furthermore, in a real‐world setting, the protocol for applying sNPWT and tNPWT may vary between healthcare settings and providers, leading to differences in clinical outcomes. Finally, confounding variables in real‐world data can introduce bias in this study. However, propensity score matching was used to balance the cohorts to control for and prevent selection bias, addressing this limitation. Propensity score matching has limitations, particularly in that it only balances observed covariates, leaving the potential for unmeasured confounding. Despite our efforts to achieve exact matches where possible, residual imbalance remained in wound area, which may have introduced some bias in the results. Additionally, the quality of matching is sensitive to the choice of calliper width; too narrow a calliper may limit the number of matches, while too wide a calliper may allow poor‐quality matches. In this study, we used the commonly recommended calliper width of 0.2, which is generally considered adequate [37].

## 6 Conclusions

This retrospective US‐based real‐world analysis found evidence of a significantly higher closure rate for all LEUs and VLUs and similar closure rates for DFUs when using sNPWT compared with tNPWT. Additionally, using sNPWT was associated with a significantly shorter time to wound closure compared to tNPWT for all LEUs. Subgroup analysis revealed a significant difference in wound closure rates for VLU, while no significant difference was observed for DFU. The overall LEU findings may be attributed to differences in the mechanisms of action between the two devices. These data provide supportive evidence for the use of sNPWT for LEUs by demonstrating the opportunity to decrease the clinical burden of LEUs on patients directly and the potential to reduce the associated financial burden of LEUs on healthcare systems.

## Ethics Statement

The authors have nothing to report.

## Conflicts of Interest

A.G. is a speaker for Smith + Nephew and AbbVie; R.L. is a paid consultant for and speaker for Smith + Nephew; L.N. is an employee of Smith + Nephew.

## Supporting information


**Table S1:** Variables used for propensity score matching.

## Data Availability

The authors have nothing to report.
