# Supplementary material for: Comparison of Single Use and Traditional Negative Pressure Wound Therapy Devices in Lower Extremity Ulcers: A US Real‐World Evidence Analysis of NetHealth Data
Source: Int Wound J. 2025 Sep 4;22(9):e70756. doi: 10.1111/iwj.70756 (PMC12411010; doi:10.1111/iwj.70756)
Supplement: Supplementary file 1 — Table S1: Variables used for propensity score matching. [file IWJ-22-e70756-s001.docx]

SUPPLEMENTARY

Supplementary Table 1. Variables used for propensity score matching.

| **Analysis** | **Variables used in the matching process** | **Variables used for exact matching** |
| --- | --- | --- |
| 1. All ulcers | Age  Gender  Ulcer area at baseline  Ulcer depth at baseline  Day of initiation of NPWT  Pressure ulcer (PU) stage (3 or 4) | Gender = F  NPWT start day >60  PU stage 3  Age >70  Region = South |
| 1. Ulcers where NPWT was initiated within 60 days | Age  Gender  Ulcer area at baseline  Ulcer depth at baseline  Day of initiation of NPWT  PU stage (3 or 4)  Diabetes mellitus  Hypertension  Cancer  Arthritis  Hyperlipidemia  Obesity | NPWT start day >60 |

**Abbreviations:** F: female; NPWT: negative pressure wound therapy; PU: pressure ulcer.
